# Magellanic penguin telomeres do not shorten with age with increased reproductive effort, investment, and basal corticosterone

**DOI:** 10.1002/ece3.3128

**Published:** 2017-06-15

**Authors:** Jack A. Cerchiara, Rosa Ana Risques, Donna Prunkard, Jeffrey R. Smith, Olivia J. Kane, P. Dee Boersma

**Affiliations:** ^1^ Center for Ecosystem Sentinals Department of Biology University of Washington Seattle WA USA; ^2^ Department of Pathology University of Washington Seattle WA USA; ^3^ School of Environmental and Forest Sciences University of Washington Seattle WA USA; ^4^ Wildlife Conservation Society The Bronx Zoo New York City, NY USA

**Keywords:** aging, comparative physiology, corticosterone, life‐history evolution, reproduction, telomeres

## Abstract

All species should invest in systems that enhance longevity; however, a fundamental adult life‐history trade‐off exists between the metabolic resources allocated to maintenance and those allocated to reproduction. Long‐lived species will invest more in reproduction than in somatic maintenance as they age. We investigated this trade‐off by analyzing correlations among telomere length, reproductive effort and output, and basal corticosterone in Magellanic penguins (*Spheniscus magellanicus*). Telomeres shorten with age in most species studied to date, and may affect adult survival. High basal corticosterone is indicative of stressful conditions. Corticosterone, and stress, has been linked to telomere shortening in other species. Magellanic penguins are a particularly good model organism for this question as they are an unusually long‐lived species, exceeding their mass‐adjusted predicted lifespan by 26%. Contrary to our hypothesis, we found adults aged 5 years to over 24 years of age had similar telomere lengths. Telomeres of adults did not shorten over a 3‐year period, regardless of the age of the individual. Neither telomere length, nor the rate at which the telomeres changed over these 3 years, correlated with breeding frequency or investment. Older females also produced larger volume clutches until approximately 15 years old and larger eggs produced heavier fledglings. Furthermore, reproductive success (*chicks fledged/eggs laid*) is maintained as females aged. Basal corticosterone, however, was not correlated with telomere length in adults and suggests that low basal corticosterone may play a role in the telomere maintenance we observed. Basal corticosterone also declined during the breeding season and was positively correlated with the age of adult penguins. This higher basal corticosterone in older individuals, and consistent reproductive success, supports the prediction that Magellanic penguins invest more in reproduction as they age. Our results demonstrate that telomere maintenance may be a component of longevity even with increased reproductive effort, investment, and basal corticosterone.

## 1 INTRODUCTION

Physiological systems deteriorate with age in most species (Austad, 2001; Haussmann and Mauck, 2008a,b; Haussmann, Winkler, Huntington, Nisbet, & Vleck, 2007; Haussmann et al., 2003; Kirkwood & Austad, 2000; Nakagawa, Gemmell, & Burke, 2004). Finding exceptions to this rule is important in understanding the physiology of aging and how it varies among species. When resources are limited, the balance between reproduction and somatic maintenance determines the rate of aging (Kirkwood & Austad, 2000; Kirkwood & Rose, 1991; Ricklefs & Wikelski, 2002). Therefore, adults must allocate resources between two competing functions: somatic maintenance and reproduction (Stearns, 1976). Studies support this conclusion, showing that increased lifetime reproductive effort reduces adult longevity as well as offspring quality (Kirkwood & Rose, 1991; Kotrschal, Ilmonen, & Penn, 2007; Ricklefs & Wikelski, 2002; Wikelski & Ricklefs, 2001) and lifespan (Boonekamp et al., 2014).

Across species, longevity is positively correlated with body mass (Haussmann et al., 2003; Lindstedt & Calder, 1976; Speakman, 2005). Generally, bird species live significantly longer than mammals of similar body size; however, understanding of the mechanisms that affect the increased longevity of birds is limited (Holmes and Austad, 1995a,b; Holmes, Fluckiger, & Austad, 2001). Even among birds, some species exceed the lifespan predicted by their mass, where predicted lifespan is based on body mass from the equation in Lindstedt and Calder (1976): lifespan *= *17.6 (mass in kg)^0.20^ (Haussmann et al., 2003). Short‐lived species, like passerines, can have low survival rates, develop rapidly, and reach sexual maturity quickly (Promislow & Harvey, 1990; Ricklefs, 2000; Saether, 1988). In long‐lived species, however, there is a fitness advantage to increased survival. While long‐lived species must allocate resources toward reproduction, they should also allocate resources to systems that enhance adult survival, like telomeres (Kirkwood & Austad, 2000; Kirkwood & Rose, 1991).

Telomeres are nucleoprotein complexes that protect the ends of chromosomes during cell replication (Blackburn, 1991). The shortening of telomere sequences correlates negatively with adult survival in most species studied to date (Bize, Criscuolo, Metcalfe, Nasir, & Monaghan, 2009; Haussmann and Mauck, 2008b; Haussmann et al., 2003; Salomons et al., 2009). During each cycle of cell replication, telomeres are shortened because DNA polymerase cannot fully replicate the 3′ end of the DNA strand (Watson, 1972); one reason telomeres shorten with age.

Telomeres shorten more slowly in long‐lived species compared to shorter‐lived species (Dantzer & Fletcher, 2015; Haussmann et al., 2003). The dynamics driving telomere shortening, however, are significantly more complex than a simple consequence of cell replication (Haussmann et al., 2003; Monaghan & Haussmann, 2006; Speakman, 2005). All vertebrates secrete glucocorticosteroids (corticosterone [CORT] in birds) from the adrenocortical cells in response to acute stress (Sapolsky, Romero, & Munck, 2000; Wingfield & Ramenofsky, 1999). Stressful events such as reproduction (Kotrschal et al., 2007), trauma and injury (Fowler, Wingfield, Boersma, & Url, 2013; Herborn et al., 2014), environmental stressors (food shortage, disturbance, storms, oiling), (Fowler et al., 2013; Walker, Boersma, & Wingfield, 2006; Walker et al., 2005a) and high‐density living (Bauch, Becker, & Verhulst, 2013; Heidinger et al., 2011; Kotrschal et al., 2007; Reichert et al., 2014; Sudyka et al., 2014) are linked to the production of stress hormones (CORT) and the shortening of telomeres (Epel et al., 2004; Haussmann, Longenecker, Marchetto, Juliano, & Bowden, 2012; Herborn et al., 2014; Kotrschal et al., 2007; Tissier, Williams, & Criscuolo, 2014). Additionally, human disturbance from tourism can increase CORT in birds (Strasser & Heath, 2011) and reptiles (French, DeNardo, Greives, Strand, & Demas, 2010). The release of CORT is correlated with reproduction. Elevated CORT can reduce both adult reproductive output and success in both penguins (*Pygoscelis adeliae* (Thierry, Ropert‐Coudert, & Raclot, 2013)) and kittiwakes (*Rissa tridactyla* (Angelier, Clément‐Chastel, Welcker, Gabrielsen, & Chastel, 2009)). Chronic increases in CORT can cause negative physiological effects including muscle degradation, as well as diminished immune function, growth, and reproduction (Johnson, Kamilaris, Chrousos, & Gold, 1992; Wingfield, 1994).

Baseline CORT levels can also be an important indicator of stress and have been shown to correlate with telomere length in a number of bird species. Increased CORT has been correlated with shorter telomeres both experimentally in long‐lived shags (*Phalacrocorax artistotlis* (Herborn et al., 2014)) and observationally in free‐living short‐lived passerine nestlings (*Aphrastura spinicauda* (Quirici, Guerrero, Krause, Wingfield, & Vasquez, 2016)). In some species, however, the correlation is not as clear. In thick‐billed murres, shorter telomeres were correlated with higher stress in years with favorable environmental conditions, and in years of poor conditions, the opposite was observed (Young, Barger, Dorresteijn, Haussmann, & Kitaysky, 2016).

Although the direct mechanistic relationship is not fully understood, telomeres may shorten in response to stress due to oxidizing reactive oxygen species (ROS) produced during increased metabolism triggered by CORT release (Beaulieu, Reichert, Maho, Ancel, & Criscuolo, 2011; Kotrschal et al., 2007; von Zglinicki, 2002). Generally, these ROS oxidize DNA bases, and the g‐rich telomeres are particularly susceptible to damaging strand breaks (von Zglinicki, 2002).

Telomere length early in life, rate of telomere shortening, and regulation of telomerase, a ribonucleic reverse transcriptase that elongates telomeres, often correlate with increased adult survival (Bize et al., 2009; Haussmann et al., 2007; Salomons et al., 2009). Telomerase is able to elongate telomeres (Greider & Blackburn, 1985); however, it often has diminished activity in adults, particularly in long‐lived species (Dong, Masutomi, & Hahn, 2005; Haussmann et al., 2007; Hornsby, 2007; Stewart et al., 2002; Tollefsbol & Andrews, 2001). There is some evidence that telomerase may be more active in bone marrow, gonads, and intestine cell lines in adult birds of long‐lived species (*Sterna hirundo; Oceanodroma leucorhoa*) compared to short‐lived ones (*Taeniopygia guttata; Tachycineta bicolor*) (Haussmann et al., 2007).

Magellanic penguins (*Spheniscus magellanicus*) live 26% longer than their predicted maximum lifespan (Boersma et al., 2013; Lindstedt & Calder, 1976). Magellanic penguins frequently lose eggs or chicks from lack of food (Boersma & Stokes, 1995; Boersma, Stokes, & Yorio, 1990), predation (Stokes & Boersma, 1998), and weather (Boersma and Rebstock, 2014). Adult penguins may also skip reproductive years if breeding conditions are suboptimal (Boersma and Rebstock, 2010a). Mechanisms that impact longevity, therefore, should be under high selection pressure, as an increase in survival would increase the probability of future reproductive opportunities if the current attempts are not successful. Our study tested whether telomere length shortened with age in Magellanic penguins and whether increased reproductive frequency and investment increase telomere shortening. We also explored the relationship between basal CORT, reproductive attempts, reproductive investment, and measures of fitness and survival. We predicted that (1) telomere lengths would shorten with age and (2) telomeres would shorten with increased reproductive effort. We also hypothesized that basal CORT would (3) correlate negatively with telomere length, (4) increase as individuals age, and (5) be higher in individuals that had to cross a tourist trail to reach their breeding nest. Finally, we predicted that older females will increase reproductive investment (6) producing larger eggs and those older adults would (7) fledge larger chicks and (8) maintain or increase reproductive success as they aged.

## 2 METHODS

### 2.1 Collection and processing

We collected blood samples from 80 known‐age adult Magellanic penguins from September to December 2007 at Punta Tombo, Argentina. We took blood from adult males of four age classes: 5 years, 15 years, 19 years, and older than 24 years (Table 1). We also collected a subset of blood from females aged 15 years (*n* = 8) to determine variation in telomere length by sex. In 2010, we took blood from 32 (28 male, four female) individuals that were sampled in 2007 to measure telomere length (Table 1). We also used our database of known‐age Magellanic penguins, beginning in 1984, to assess trends in age, reproductive investment, and chick survival (*n* = 975).

**Table 1:** Age groups and sample size

Age (2007)	*n*	Age (2010)	*n*
5 years (Male)	15	8 years	7
15 years (Male)	17	18 years	12
15 years (Female)	8	18 years	4
19 years (Male)	15	22 years	5
≥24 years (Male)	18	≥27 years	4
Total	73	–	32

We know the age of adults 19 yo and younger because they were banded as chicks (year 0) or as juveniles (year 1). Penguins in the oldest adult cohort, aged 24 years or more, were banded as adults in 1983. The sex of the adults were known prior to sample collection (Boersma & Davies, 1987). Sex is known from morphological traits (Boersma et al., 2013), and this method is accurate for >97% of adults (Bertellotti et al., 2002).

### 2.2 Sample collection and DNA extraction

We used a 22‐ to 25‐gauge hypodermic needles to collect blood from a vein on the dorsal surface of the foot, distal to the tarsometatarsus. Blood was collected into a heparinized capillary tube (Thermo Fisher Scientific Inc.). We collected blood within 3 min of sighting the bird and released them within 5 min of capture. We immediately placed the blood into anti‐lysis buffer (10% DMSO/90% newborn bovine serum). We also collected whole blood into a separate, empty Eppendorf 1.5‐ml microcentrifuge tube. The tube containing whole blood was centrifuged for 10 min at 1000–2000 g (3–5k rpm) at room temperature (~20–22 °C), and the plasma supernatant removed and placed in a third clean tube. We froze all samples at −18 °C within 1 hr of collection. Samples were stored at −18 °C for approximately 2–6 weeks depending on collection date, and flown to the United States on a combination of dry ice and blue ice packs. After transporting samples to the United States, we stored the samples at −80 °C at the University of Washington, until processing.

We extracted DNA from a lightly centrifuged cell pack, consisting primarily of erythrocytes (Qiagen DNeasy Mini‐kit), and then quantified DNA via NanoDrop spectrophotometer (mean 260/280 ratio ± *SE* = 1.85 ± 0.02). We measured telomere length by quantitative polymerase chain reaction (qPCR), as in Cerchiara et al. (2017).

### 2.3 Quantitative polymerase chain reaction

Briefly, we ran two PCRs for each sample, based on the method described by Cawthon (2002). The first PCR amplified the telomeric DNA and the second amplified a single‐copy control gene (36B4, acidic ribosomal phosphoprotein PO). The control gene PCR is used to normalize the starting amount of DNA. A melting (dissociation) curve was run at the end of every PCR to confirm the presence of a single amplification product (Ringsby et al., 2015). We selected a random subset of samples (*n* = 20) to verify the 36B4 primers created a single repeatable product for the control gene. Our primers targeted the 75‐bp oligomer of the 36B4 reference gene. All samples showed the same product (at ~75 bp) when ran on a 2% gel, and no secondary products, so we are confident in the accuracy of our control gene (O'Callaghan & Fenech, 2011).

We included a four‐point standard curve (twofold serial dilutions of a high‐quality adult male DNA sample from 10 to 1.25 ng of DNA) in all PCRs to allow the transformation of raw *C*_t_ (cycle threshold) into nanograms of DNA and quantify assay efficiency (mean telomere efficiency ± *SE* = 0.74 ± 0.01, mean 36B4 efficiency ± *SE* = 0.88 ± 0.02; all *R*_sq_ > .99). In each trial, two control samples were run to allow for normalization and reproducibility trials to confirm correct measurements. The intratrial variability and intertrial variability (coefficient of variation) for the qPCR were 7% and 8%, respectively, which is typical for this assay (Cawthon, 2009; Martin‐ Ruiz et al., 2014).

### 2.4 Measurement of basal corticosterone

We measured the CORT in the plasma at the University of Washington using radioimmunoassay using 125I CORT RIA kits (#07‐120103; MP Biomedicals, Costa Mesa, CA).

We performed parallelism and accuracy validations on the radioimmunoassay using a plasma pool to ensure the antibodies recognize the CORT in a predictable manner and did not exhibit interference. This validation was performed by serially diluting a pool of plasma samples with steroid diluent provided with the kit. We then assayed each of those dilutions separately. We determined which dilution was closest to 50% binding and selected that dilution to perform a spiked recovery test (accuracy). We mixed known concentrations of CORT with the pooled sample dilution and measured the recovery to determine the accuracy of the analysis.

We then diluted all samples to 1:15 based on the parallelism and accuracy validations. We reported hormone values as nanograms per milliliter based on the values measured in the assay multiplied by the dilution factor. Intra‐assay variation and interassay variation was 2.8% and 3.3%, respectively.

### 2.5 Statistical analysis

To test whether telomere length was predicted by age, we used a linear model where telomere length was regressed on age in 2007 (*n* = 73, Table 1). Because telomere length among males and females aged 15 years (*t* test) was similar, the data were pooled. Cross‐sectional data have a particular limitation when examining telomere length over age. It is possible that birds of low quality, and potentially shorter telomere length, die earlier. Therefore, only those high‐quality birds with long telomeres survive and are sampled, presenting the appearance of telomere maintenance or even elongation (Haussmann and Mauck, 2008b). It is for this reason our longitudinal analysis of resampling birds is critical to determining telomere dynamics.

We defined telomere rate of change (TROC) to be = (2010 telomere length—2007 telomere length) ÷ 3 years. We asked whether telomere length changed for individuals over the 3 years 2007–2010. As the change in age for all individuals was 3 years, and TROC did not vary by age, we compared each bird's telomere length in 2007 to its telomere length in 2010. As all individuals were resampled, we used a paired *t* test to compare means, an accepted technique when comparing telomere lengths of birds resampled longitudinally (Pauliny, Wagner, Augustin, Szep, & Blomqvist, 2006). Also, as all birds were 3 years older when resampled, we asked whether telomere length had shortened in 3 years. To test this, we used a linear regression where TROC is predicted by mean age of the individual, or 2007 age plus 1.5 years.

Next, we assessed the effect of reproductive effort on telomere length for all individuals sampled in 2007. Magellanic penguins at Punta Tombo breed synchronously with most first eggs laid within a 2–3 week period in early October (Boersma and Rebstock, 2014; Boersma et al., 1990). We found telomere length for individuals that hatched one or two eggs, or fledged one or two chicks, was similar so we considered a year when at least one egg was laid, a year of reproductive effort. To be more confident that we had an accurate measure of breeding attempts for an individual, we included in our analysis only penguins sighted as adults in the colony before they were 7 years of age, as both sexes usually breed by that age, if they have a mate (Boersma et al., 2013). We regressed the total number of years breeding during that individual's lifetime against the individual's age and computed the residuals. These residuals were regressed against telomere length, with the added factor of sex. We also used a linear model to ask whether previous reproduction (number of chicks fledged) prior to 2007 predicted the TROC between 2007 and 2010. We checked whether telomere length in 2007 was correlated with the TROC with a Spearman rank correlation. As they were negatively correlated, we included telomere length in 2007 as a covariate, as in Beaulieu, Ropert‐Coudert, Le Maho, Ancel, and Criscuolo (2010).

Next, we included only samples for which we collected blood in both 2007 and 2010 (*n* = 33) in a general linear model. In this model, reproductive success (*chicks fledged/eggs laid*) during the 3 years 2007–2010 predicted TROC, with mean age and 2007 telomere length included as covariates. Subsequent models tested whether the number of reproductive attempts (years of breeding), number of eggs laid, or the number of chicks fledged from 2007 to 2010 predict the TROC during the same period.

Corticosterone concentration (ng/ml) was log‐transformed for all analyses. Samples were collected between 0730 and 1900. We used a linear regression to test for the relationships between CORT, age, date, time of collection, and telomere length, as well as the two‐way interaction terms. We conducted backwards simplification using the “step” function in R with a maximum of 1000 iterations (R v3.1.3). The “step” package simplifies models based on Akaike information criteria (Aho, Derryberry, & Peterson, 2014). The function is conservative by nature, retaining *p*‐values as high as .33, and removed time as a significant variable. This function returned significant correlations between CORT, date and age, and the first‐order interaction between date and age. We used a linear model controlling for age and date of collection to test whether CORT predicts telomere length. We also used a linear model to test whether basal CORT was predicted by age, controlling for the date of collection. Finally, we used a linear model to determine whether basal CORT was predicted by the date of collection, with age as a covariate.

We used GPS coordinates for breeding nests in 2007, and separated nests based on whether the adult penguin had to cross a tourist trail to reach their nest from the sea. This tourist trail is walked by over 100 k tourists annually (Boersma, 2008). We used a *t* test with unequal variances to test for higher basal CORT in penguins that had to cross the trail.

We also measured reproductive investment, as volume and mass of eggs in a clutch and the mass of chicks fledged in a season. We tested whether older females laid larger eggs than younger females. We used a mixed‐effects model that included penguin ID as a random effect to control for repeated measures of the same individuals. We included age squared to allow for a quadratic relationship. Total egg size was the sum of egg volumes for 975 clutches of known‐age females where egg volume for the first laid egg is volume = 1.6996 + 0.4967 × length × width^2^, and for the second eggs is volume = 8.2723 + 0.4758 × length × width^2^ (Boersma and Rebstock, 2010b).

We used a linear mixed‐effects model to determine the correlation between fledgling mass and age of the parent. We included only those nests that fledged two chicks and took the maximum weight for each chicks measured after January 9 and before March 1st of the season. Penguin chicks that weighed 1,800 g after January 9 and are not found dead generally fledge (Boersma et al., 1990). We included bird ID and year as random effects to control for the repeated measures of individual birds, and the year in which the bird bred. Sex and total clutch volume were included as fixed effects.

We also used a linear mixed‐effects model to determine the correlation between yearly reproductive success (*chicks fledged/eggs laid)* and the age of the parent. Here, we included bird ID and year as random effects and sex and clutch volume as fixed effects.

Next, we used a binomial generalized linear model to determine whether fledging weight predicted resighting of the chicks as adults in the colony. We used the same chick weight as was used for the other analyses and included hatch order (1 or 2), year, and their two‐way interaction as a predictors in the model.

For statistical tests, we used R Statistical software (R Foundation for Statistical Computing: Development Core Team (v3.1.3).

## 3 RESULTS

### 3.1 Telomeres and age

**Figure 1:**
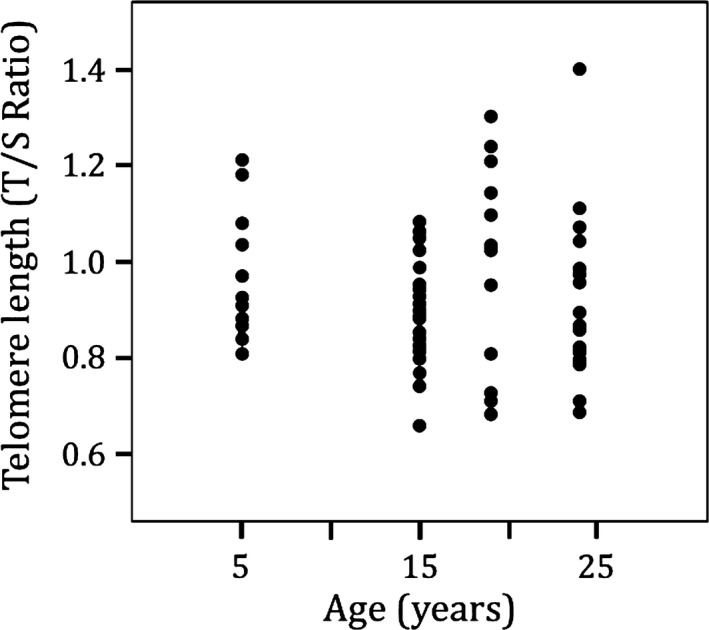
Telomere length is similar in adult Magellanic penguins aged 5 to older than 24 years of age. Telomere lengths for the penguins collected in a cross‐sectional sample in 2007 showed no relationship with age, suggesting high telomere maintenance through 24+ years (*R*_sq_ < .001, *p *=* *.9156, *n *=* *73). Telomere length is the relative telomere (T) to single copy gene (S) ratio

**Figure 2:**
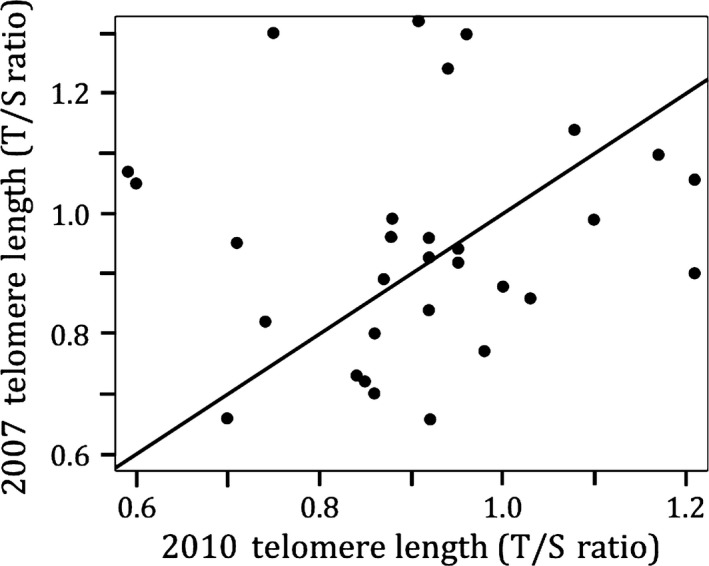
Longitudinal sampling of adults over a 3‐year period shows no telomere change, regardless of age. Regardless of age, telomere length of Magellanic penguins did not shorten over a 3‐year period; line is *x* = *y* (*t* = 0.95, *p* = .34, *n* = 32). Telomere length is the relative telomere (T) to single copy gene (S) ratio

Telomeres of adult male and female Magellanic penguins 15 years of age were similar in length, so we pooled them in subsequent analyses (*t* = 0.44, *p* = .66, *n* = 25). Telomere lengths for the 73 penguins sampled in 2007 were similar among age groups (*R*_sq_ < .001, *p* = .92, *n* = 73, Figure 1). In this test, age was included as a factor, although testing as a continuous variable yielded the same result (*p* = .83). The telomere lengths of 32 adults sampled 3 years later had not changed significantly in length (*t* = 0.95, *p* = .34, *n* = 32; Figure 2). Telomere rate of change (*telomere length change/year*) did not correlate with mean age of individual, suggesting that telomere attrition rate does not increase with age (*R*_sq_ = .06, *p* = .57, *n* = 32).

### 3.2 Telomeres and reproduction

**Figure 3:**
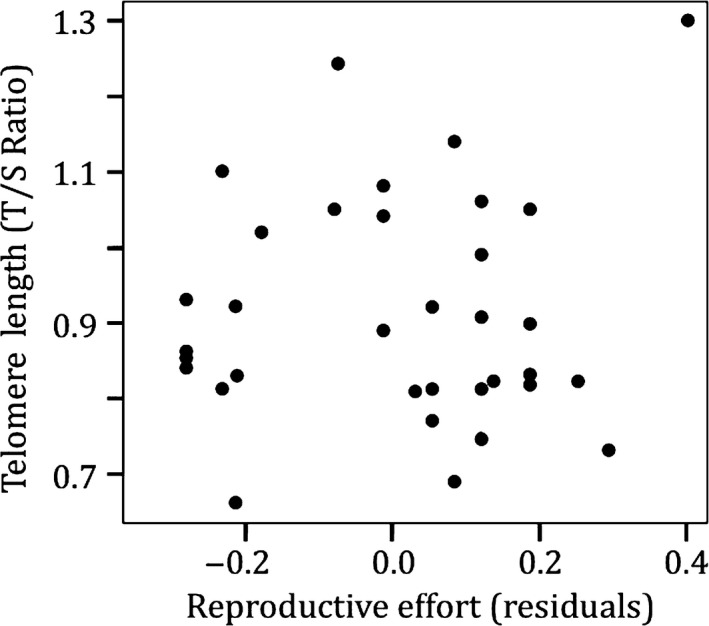
Telomere lengths of adult Magellanic penguins are not predicted by reproductive effort. Telomere length is the relative telomere (T) to single copy gene (S) ratio. Linear model of residual values for the number of reproductive attempts by age of individuals regressed against telomere length, controlling for sex of individual (*R*_sq_ = .027, *p *=* *.663, *n *=* *37)

Reproductive years did not predict telomere length for penguins aged 15 yo and 19 yo, even when we controlled for sex (*R*_sq_ = .027, *p* = .66, *n* = 37, Figure 3). Additionally, the number of chicks fledged prior to 2007 did not predict the TROC from 2007 to 2010 (*p* = .86, *n* = 33). Likewise, the number of eggs laid (*p* = .76), chicks fledged (*p* = .60), number of reproductive years (*p* = .73), or reproductive success (*p* = .24) during 2007–2010 did not predict TROC during the same period (*n* = 33).

### 3.3 Corticosterone

**Figure 4:**
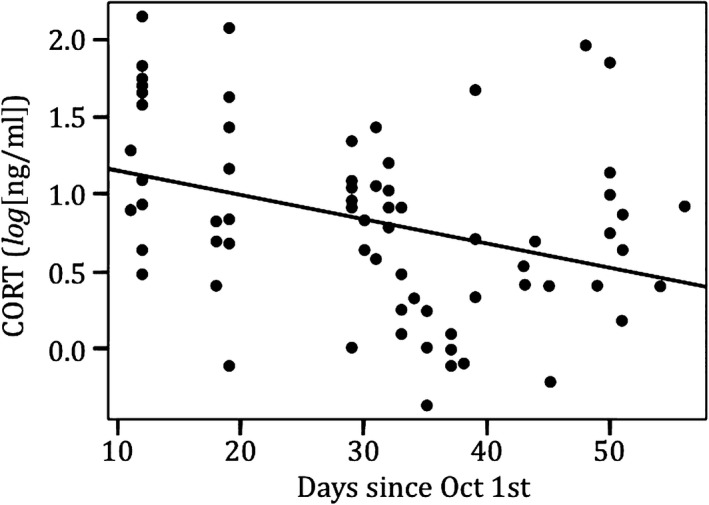
Corticosterone deceases during the breeding season. During the breeding season, corticosterone (log(ng/ml)) was higher earlier and decreased during the season, controlling for age of individual (*R*_sq_ = .22, *n* = 70, *p* = .015). Corticosterone concentration (ng/ml) was log‐transformed. Samples were collected from October–November, so we used days since October 1st as a continuous variable

**Figure 5:**
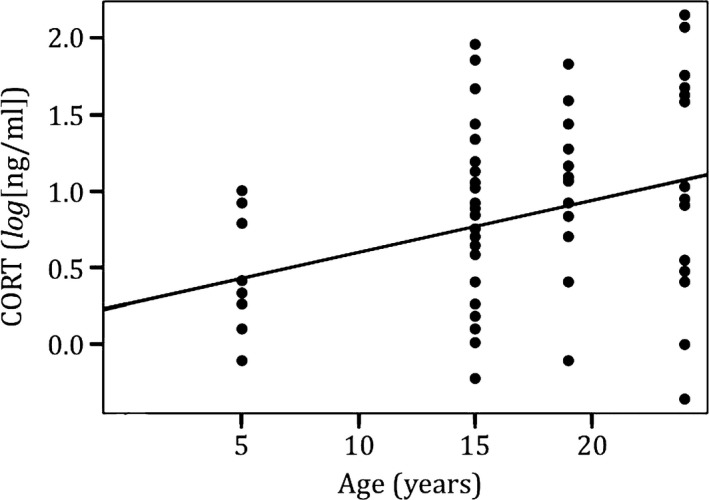
Corticosterone increases in older adult Magellanic penguins. Corticosterone was positively correlated with age of adult penguins, controlling for time of breeding season (*R*_sq_ = .22, *n* = 70, *p* = .004). Results are similar when age is treated as continuous or as a factor. Corticosterone concentration (ng/ml) was log‐transformed

Corticosterone was not correlated with telomere length in adults, when we controlled for age and date of collection (*R*_sq_ = .07, *n* = 64, *p* = .34). Corticosterone was higher earlier in the season and decreased with time through the end of November, when controlling for age (*R*_sq_ = .22, *n* = 70, *p* = .015; Figure 4). Basal CORT was positively correlated with age of adult penguins, when controlling for date of collection (*R*_sq_ = .22, *n* = 70, *p* = .004; Figure 5). Finally, we found that having to cross a tourist trail to reach breeding nest did not significantly increase the basal CORT of penguins (*t* = 0.9097, *n* = 67, *p* = .36).

### 3.4 Age, reproductive investment, and chick survival

We found that middle‐aged females produced the largest eggs. A quadratic function total clutch volume ~ age + age^2^ best fit this relationship (*R*_sq_ = .08735, *n* = 975, *p* < .001). Total clutch mass at fledging was similar for both sexes, controlling for age, year, clutch volume, and individual (*t* = 1.952, *n* = 211, *p* = .0528). Total clutch volume was significantly correlated with total fledgling mass for two‐egg and two‐chick clutches (*t* = 3.26, *n* = 211, *p* = .001).

Total fledgling mass was not correlated with age in males and females (*t* = −0.590, *n* = 211, *p* = .556). The covariate total egg volume was significant (*t* = 3.265, *n* = 211, *p* = .001), but sex was not (*t* = 1.952, *n* = 211, *p* = .053). Reproductive success was not correlated with age in adult penguins when controlling for sex, year, and penguin ID (*F* = 1.48, *n* = 372, *p* = .22).

When controlling for hatch order and year, we also found that chicks that returned as adults fledged at a significantly heavier weight than those that did not (*z* = 1.98, *n* = 5,996, *p* = .048). First‐hatched chicks were seen as adults more often than second‐hatched chicks (*z* = −2.7, *n* = 5,996, *p* = .007).

## 4 DISCUSSION

Telomeres of Magellanic penguins were of similar length for adults aged 5–24 years of age, which contrasts with the classical theory that telomeres shorten with age (Bize et al., 2009; Haussmann and Mauck, 2008a; Haussmann et al., 2003; Salomons et al., 2009). Similar phenomena has been shown in other long‐lived adult birds and reptiles, including the long‐lived Barnacle goose (*Branta leucopsis*) (Pauliny, Larsson, & Blomqvist, 2012), Wandering albatross (*Diomedea exulans*), European shag (*Phalacrocorax Aristotelis*) (Hall et al., 2004), and Leatherback turtle (*Dermochelys coriacea*) (Plot, Criscuolo, Zahn, & Georges, 2012). Previous studies show that telomere shortening is correlated with decreased survival (Bize et al., 2009), reduced likelihood of return (Salomons et al., 2009), and lower lifelong reproductive success (Pauliny et al., 2006), even with adult maintenance of telomeres (Salomons et al., 2009), so there may be a survival advantage to longer telomeres.

The longitudinal analysis is a critical aspect of our study as selective mortality of less fit individuals could appear as telomere maintenance (Haussmann and Mauck, 2008b). We show, however, telomeres did not shorten over a 3‐year period, regardless of the age of the individual. It should be noted that we did not assess female telomere change in 5‐, 19‐, or 24+‐year‐old birds, so we cannot conclude sex‐specific changes in telomere length for those females. It is also possible that the measurement error of the assay may not capture telomere shortening if only few base pairs are lost in 3 years.

We found that CORT, a stress hormone, correlated with the date of collection during the season. CORT was higher for individuals in October, at the beginning of the breeding season, and decreased until the end of November, when chicks begin to hatch. Magellanic penguins arrive at the breeding colony in mid‐September, and males arrive a few weeks before females to secure nesting sites and compete with other males for nests, which is when CORT was the highest (Boersma et al., 2013). Penguins have long fasting periods before egg laying and during incubation, have antagonistic interactions with conspecifics, and defend offspring, and females lay two eggs that require nutritional investment (Boersma et al., 2013). After this period, Magellanic penguins assume biparental care exchanging bouts of fasting and foraging during incubation and chick rearing. Magellanic penguin CORT decreased during the egg incubation period, and continued to decrease until mid‐November, during which time chicks begin to hatch and competition for mates and nest sites declines in males. High CORT early in the reproductive cycle followed by a decrease is the general pattern observed in other birds (Piersma, Reneerkens, & Ramenofsky, 2000; Reneerkens, Guy Morrison, Ramenofsky, Piersma, & Wingfield, 2002).

While CORT response to stressors might affect telomeres in other species, Magellanic penguins generally have a little variation in baseline CORT or corticosteroid stress response (Walker, Boersma, & Wingfield, 2015). Also, Magellanic penguins that had been fighting do not increase CORT release from baseline (Walker et al., 2015), unlike losers of fights in other species (Oyegbile & Marler, 2006; Schuett & Grober, 2000; Verbeek, Iwamoto, & Murakami, 2008). Fasting Magellanic penguins also did not elevate baseline CORT until they fasted for several weeks (Hood, Boersma, & Wingfield, 1998). We also found that having to cross a tourist footpath to reach their nest when entering or leaving the colony did not have increased basal CORT. In adults, penguins that lived near tourist areas had a diminished CORT response to capture stress than those living in more isolated areas (Fowler, 1999; Walker et al., 2006). Additionally, individuals living in more isolated areas had higher basal CORT than those living in tourist areas (Fowler, 1999). Likewise, fledglings living with tourist visitation did not secrete more CORT when captured (Walker et al., 2005b). These results suggest penguins likely habituate to these chronic stressors, which may help mitigate the damage to maintenance systems. These finding are all consistent with Magellanic penguin's having low baseline CORT and being resistant to stress which may be a component of why their telomeres do not shorten.

Diet may also play a role in telomere maintenance, likely by bolstering the antioxidant ability of blood plasma, a hypothesis proposed for other penguin species (Beaulieu et al., 2010, 2011). Stressors can increase oxidative stress (Alonso‐Alvarez et al., 2004), which can shorten telomeres (von Zglinicki, 2002). However, penguins may have a better ability to neutralize ROS through antioxidant defense than other bird species or mammals. Antioxidants present within the blood plasma are, in part, a result of the intake of prey that possess high antioxidant load (Cohen, McGraw, & Robinson, 2009). Adelie penguins (*P. adeliae*) can preferentially feed within prey assemblages that consist primarily of krill (*Euphausia superba* and *E. crystallorophias*), a high antioxidant prey, in response to stress (Beaulieu et al., 2010). Magellanic penguins at Punta Tombo feed upon fish, squid, and crustaceans (Boersma et al., 2013). Squid (*Loligo* spp., *Illex argentinus*) is an important component of their diet, and can account for 1%–19% of prey observed, observed in stomach contents (Gandini et al., 1994; Wilson et al., 2005). A diet of just 2% squid ink significantly increased the antioxidant ability in chickens (*Gallus gallus*) over a 42‐day study (Liu, Luo, Chen, & Shang, 2011). The diet of Magellanic penguins may help mitigate the effects of ROS increases and minimize shortening of telomeres by bolstering their antioxidant ability.

We found no relationship between an individual's reproductive effort and telomere length, or the TROC. These results are consistent with results from other penguin species. Beaulieu et al. (2011) showed that Adelie penguins with experimentally increased reproductive effort, by means of handicapping, did not have significantly shorter telomeres (Beaulieu et al., 2011). We also show that number of reproductive attempts was not correlated with telomere length for penguins aged 15 yo and 19 yo. While the relationship between increased reproductive effort and telomere length is well studied, our results are in contrast to most studies. Experimentally increased reproductive effort is correlated with increased telomere shortening in both laboratory studies of zebra finch (*T. guttata*) (Heidinger et al., 2011; Reichert et al., 2014) and wild blue tits (*Cyanistes caeruleus*) (Sudyka et al., 2014). Common terns (*S. hirundo*) with increased reproductive output (larger brood sizes) also had shorter telomeres (Bauch et al., 2013). However, individuals with the highest reproductive success showed the smallest shortening of telomeres (Bauch et al., 2013), suggesting that long‐lived birds of higher quality may better mitigate the cost of reproduction. It may be possible, however, that our results stem from a variation in investment and high‐quality birds that may possess more resources to invest more in both maintaining telomeres and reproductive effort. Magellanic penguins appear to be able to mitigate shortening of their telomeres, although individual quality, body condition, or behaviors related to chick rearing may be important in reducing this shortening.

Consistent with life‐history theory, young to middle‐aged females allocate more resources to reproduction as they age. The late‐life decline we observed may be explained by reproductive senescence. These birds that maintain telomeres despite increased egg volume may be of high quality and able to invest in both maintenance and reproduction. They also have heavier fledglings and maintain their reproductive success (*chicks fledged/eggs laid*) as they age. This increased egg size also increases fledging success (Reid & Boersma, 1990). We also found heavier fledglings were more likely to be seen as adults, suggesting a fitness advantage to fledging at a higher weight. In other species of birds, older adults produce increased clutch size (number of eggs) and fledge more chicks even when laying eggs later in the breeding season (Forslund & Larsson, 1992). This is likely a combination of increased resource allocation to reproduction as well as increased foraging effort or efficiency, a measure of individual quality. Previous studies support the latter as higher‐quality penguins also tend to fledge heavier chicks even when experimentally given smaller eggs (Reid & Boersma, 1990). Basal CORT, however, was also positively correlated with age of adult penguins, suggesting either aging penguins are allocating more resources toward reproduction, or reproduction is more taxing. Increases in CORT can cause increased mobilization of energy stores and gluconeogenesis (Greenberg & Wingfield, 1987; Wingfield et al., 1998). Therefore, the increase in CORT in older penguins could suggest that aging adults may be allocating resources to increase the probability of successful reproduction, maintaining fitness as they age.

The physiological mechanisms that govern aging are complex, and adult maintenance of telomeres is only one component of penguin longevity. Our results demonstrate that despite increases in CORT and increased investment in reproduction, telomeres are maintained in Magellanic penguins, and this may be a component of longevity in long‐lived species.

## CONFLICT OF INTEREST

None declared.

## AUTHOR CONTRIBUTIONS

JAC and PDB designed the research; JAC, RAR, DP, JRS, and OJK performed the research; PDB, RAR, and DP contributed reagents and analytical tools; RAR and JAC analyzed data; JAC wrote the manuscript; and JAC, RAR, DP, JRS, OJK, and PDB assisted in editing the manuscript.

## DATA ACCESSIBILITY

The data for this study are archived at the Center for Ecosystem Sentinels at the University of Washington, Seattle, WA, USA.
